# Pre- and post-COVID-19 pandemic identification of dengue hotspots and exploration of population and environmental determinants of dengue in Quezon City, Philippines

**DOI:** 10.1186/s41182-025-00789-3

**Published:** 2025-08-14

**Authors:** John Robert Carabeo Medina, Shin’ya Kawamura, Rie Takeuchi, Rolando V. Cruz, Johnedel Mendoza, Paul Michael R. Hernandez, Fernando B. Garcia, Ernesto R. Gregorio, Jun Kobayashi

**Affiliations:** 1 Institute of Clinical Epidemiology, National Institutes of Health, University of the Philippines Manila, 623 Pedro Gil St, Ermita, Manila, 1000 Metro Manila, Philippines; 2 Department of Clinical Epidemiology, College of Medicine, University of the Philippines Manila, 547 Pedro Gil St, Ermita, Manila, 1000 Metro Manila, Philippines; 3 National Institute of Technology, Matsue College, 14-4 Nishi-Ikumacho, Matsue, Shimane 690-8518 Japan; 4 Graduate School of Public Health, International University of Health and Welfare, 4-3, Kodunomori, Narita, Chiba 286-8686 Japan; 5 Quezon City Epidemiology and Surveillance Division, Quezon City Health Department, Local Government of Quezon City, Quezon City, Philippines; 6 Department of Environmental and Occupational Health, College of Public Health, University of the Philippines Manila, 625 Pedro Gil St, Ermita, Manila, 1000 Metro Manila, Philippines; 7 Healthy University Office, University of the Philippines Manila, Manila, Philippines; 8 Department of Health Policy and Administration, College of Public Health, University of the Philippines Manila, 625 Pedro Gil St, Ermita, Manila, 1000 Metro Manila, Philippines; 9 Department of Health Promotion and Education, College of Public Health, University of the Philippines Manila, 625 Pedro Gil St, Ermita, Manila, 1000 Metro Manila, Philippines; 10 Department of Global Health, Graduate School of Health Sciences, University of the Ryukyus, 207 Uehara, Nishihara-cho, Nakagami-gun, Okinawa, 903-0215 Japan

**Keywords:** Dengue, Philippines, Geographic information system, GIS, Disease hotspot, Quezon City, COVID-19

## Abstract

In 2020, there were decreasing trends in reported dengue cases in several countries in the Western Pacific Region, including the Philippines, amid the COVID-19 pandemic. In this study, the spatial distribution of dengue incidence and the hotspots were described quarterly in Quezon City from 2019 to 2022, with the unit of analysis being the 142 *barangays* (small territorial and administrative villages) in Quezon City. The influence of selected environmental and demographic factors on the persistence of dengue was also explored. Surveillance data from the Quezon City Epidemiology and Surveillance Division, Normalized Difference Vegetation Index (NDVI), and satellite images of built environments, such as buildings, facilities, and transportation network hubs were processed and analyzed. The local Moran’s I statistics performed with 95% confidence level was employed in the identification of local dengue hotspots. Dengue hotspot maps and maps of greenness, surrounding greenness, small building ratios, and the number of transportation network hubs in each barangay (village) of Quezon City were developed. The association of dengue cases with greenness, surrounding greenness, transportation network hubs, small building ratios, and population density was determined through a generalized linear model (GLM). Results revealed that incidence rates of dengue across *barangays* were spatially heterogeneous, and the dengue hotspots were unstable as they varied quarterly each year. Proximity to surrounding greenness (NDVI 1 km), small building ratio, and presence of transportation network hubs were found to be significantly associated with dengue hotspots. Dengue prevention strategies, such as search-and-destroy activities for breeding sites, are suggested to be implemented regularly in such areas. The study also recommends considering the anticipated increase in dengue hotspots and the significant environmental factors in the development of health promotion and education campaigns.

**Supplementary Information:**

The online version contains supplementary material available at 10.1186/s41182-025-00789-3.

## Introduction

Dengue is a mosquito-borne viral disease with varying clinical presentations from a mild, febrile illness to hemorrhagic fever and shock syndrome [1]. It is caused by any of the four genetically related serotypes of the dengue virus, which belongs to the genus Flavivirus of the Flaviviridae family [2]. Primary infection with one serotype does not confer lifelong immunity to other serotypes, and secondary infection with another serotype increases the risk for severe disease [3]. Infected *Aedes aegypti* and *Aedes albopictus*, which are ubiquitous in tropical and subtropical regions, influence the geographical distribution of the disease and perpetuate the transmission cycle of the virus among humans, referred to as urban transmission [4, 5].

Over the past two decades, a tremendous increase in reported dengue cases was noted in 129 countries [6]. The global incidence of dengue surged tenfold from 505,430 cases in 2000 to 5.2 million in 2019, but modeling studies suggested that this could be underestimated [7, 8]. Remaining uncontrolled worldwide, efforts to mitigate this neglected tropical disease warrant more political support and attention in terms of research and development [9].

In the countries of World Health Organization’s Western Pacific region, dengue persists as a public health problem. Analysis of regional surveillance data from 2013 to 2019 revealed that the annual number of dengue cases in the region increased from 430,023 in 2013 to 1,050,285 in 2019, while the case fatality ratio ranged from 0.19 to 0.30% during the period [10]. The active co-circulation of the different serotypes of the dengue virus in most countries causes the hyperendemicity of the disease in the region and the occurrence of large-scale outbreaks [11, 12]. Unfortunately, most member countries in the region were prominent tourist destinations, which could facilitate the importation of dengue in non-endemic areas. Dengue cases imported from these countries were reported not only in Europe and the United States, but also in some non-endemic countries in the Western Pacific region. Almost all reported dengue cases in Japan, Korea, and Australia from 2016 to 2018 were imported cases [10]. These reports prove that global travel had exposed travelers to an increased risk of dengue infection and facilitated transmission across national borders. Asian countries such as Thailand, Indonesia, and the Philippines were documented as the major sources of imported dengue in Europe and the United States [13]. Megacities in these countries, which include Bangkok, Jakarta, and the cities in Metropolitan Manila, were known hyperendemic areas [14–16]. These places have long been carrying the burden of dengue, as perpetuated by urbanization, unsanitary conditions, and the presence of mosquito vectors 13. Moreover, transportation networks could facilitate intra-urban mobility of both vectors and infected cases, which would promote vector expansion and disease transmission. A study in Bangkok showed that the density of public transportation stops or terminals is dengue hotspots [17].

During the height of the COVID-19 pandemic, particularly in 2020, decreasing trends in reported dengue cases were noted in several countries in the Western Pacific region, including the Philippines [18]. While the drop in the number of reported dengue cases could be a consequence of reduced transmission due to restrictions in human mobility amid community quarantines and lockdowns, people’s fear of contracting COVID-19 in health facilities thereby influencing their health-seeking behavior, as well as the diversion of the health system’s attention and resources towards COVID-19 response, were also plausible factors to take into account. All of these had contributed to underreporting of dengue cases [19].

Some proof-of-concept on the potential use of disease mapping, spatial analysis, and hotspot identification in understanding the epidemiology of dengue had already been demonstrated in some localities in the Philippines, including Quezon City [20–22]. Quezon City, which is one of the highly urbanized cities in Metropolitan Manila, also known as the National Capital Region (NCR), has long been facing dengue as a persistent public health concern [20, 23]. Previous spatial epidemiological studies on dengue in Quezon City employed geographic information system mapping and/or local cluster detection using Getis-Ord Gi* statistics [20, 23]. However, the influence of environmental factors on the persistence of dengue in the city was only hypothesized, if not investigated, in those studies [20–23]. Moreover, those studies analyzed annual dengue data. Hence, this study was conceptualized to address these research gaps.

In this study, the spatial distribution of dengue incidence was described quarterly every year from 2019 to 2022. Dengue hotspots were also identified every quarter in a year. The influence of selected environmental and population factors on the magnitude of dengue cases was also explored. Specifically, the environmental factors that were investigated were greenness, surrounding greenness, small building ratio, and transportation network hubs, while population density was the sole population factor that was assessed. The influence of these factors on dengue had already been explored in studies done in other countries, but it remains open for exploration in the Philippine context [24–27]. To the best of the authors’ knowledge, this is the first study that has investigated the association of those indicated environmental and population factors with the magnitude of reported dengue cases in Quezon City. The findings of this study will be useful in developing timely and tailored-fit policies and program interventions that can be implemented to address the continuing problem of dengue in Quezon City. Moreover, this study itself is evidence of the utility of GIS and hot spot analysis that can be adapted in the routine practice of dengue surveillance. Lastly, the analysis of the association of environmental and population factors could provide new insights on the drivers of dengue transmission.

## Methods

### Study area

**Fig. 1:**
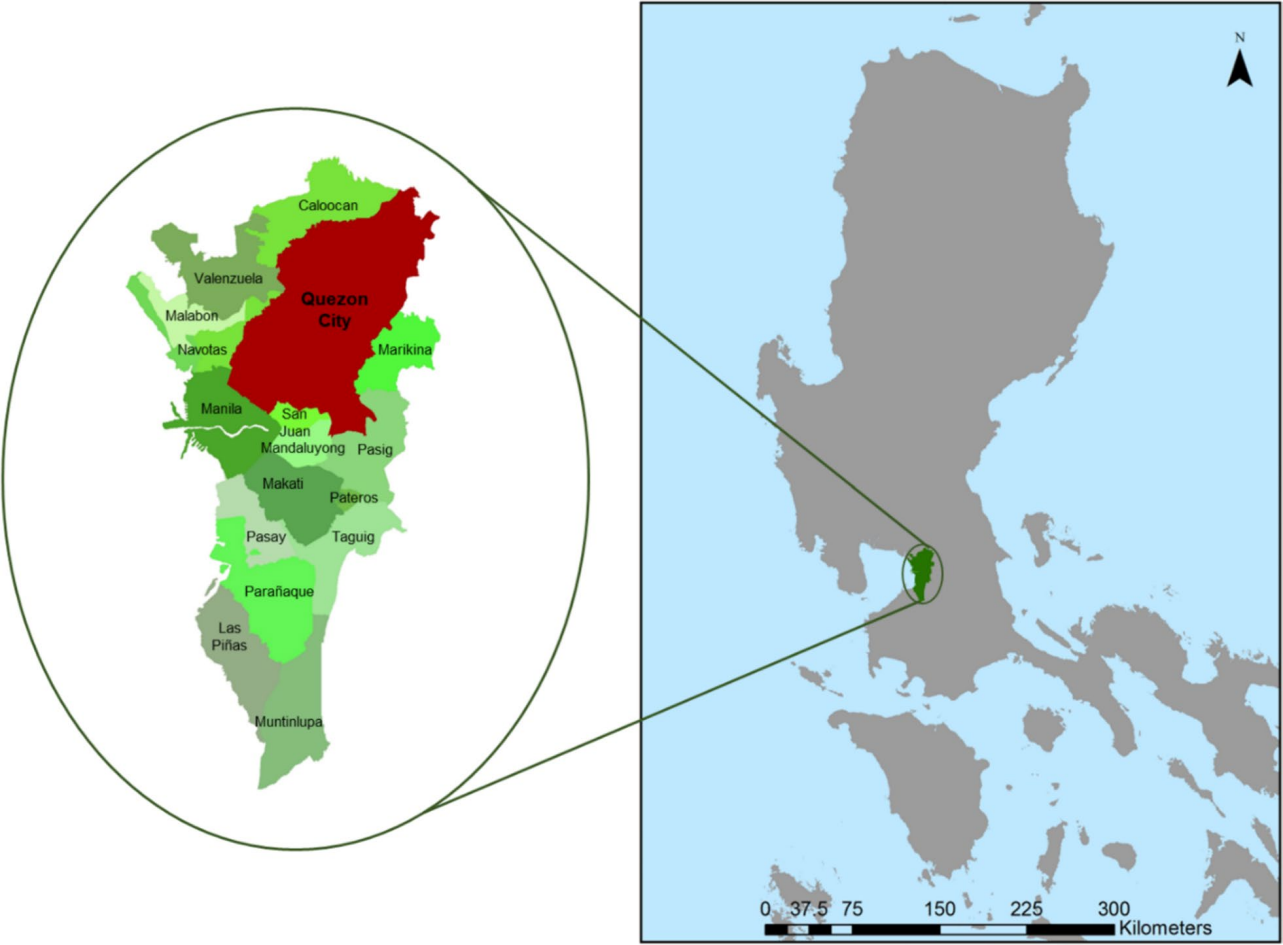
Locator map of Quezon City in the National Capital Region, Philippines. Quezon City is one of the 16 highly urbanized cities in the National Capital Region

The study was implemented in Quezon City, one of the cities in the NCR in the Philippines. Situated in the northeastern part of the NCR, it is bounded in the west by the cities of Caloocan, Valenzuela, and Manila; in the south by the cities of Manila, San Juan, Mandaluyong, and Pasig; and in the east by the cities of Pasig and Marikina, and the municipalities of San Mateo and Rodriguez in Rizal province; and in the north by San Jose del Monte City in Bulacan and some portions of Rodriguez in Rizal (Fig. 1) [28].

**Fig. 2:**
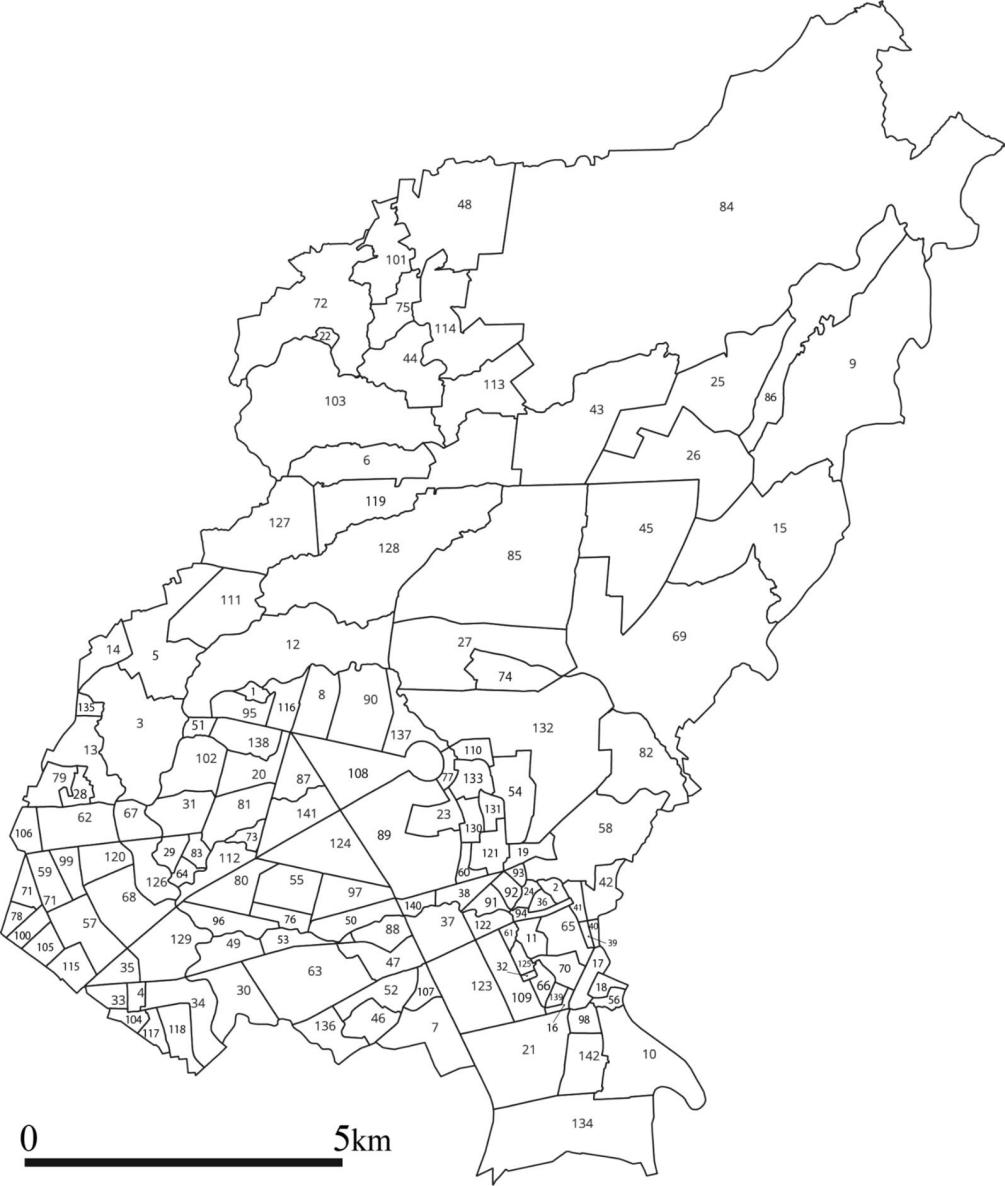
Six legislative districts and *barangays* of Quezon City, National Capital Region, Philippines. Quezon City is subdivided into 142 *barangays*, which are grouped into six legislative districts (Additional file 1)

With a land area of 171.1 sq. km, Quezon City covers one-fourth of the total land area of NCR [29]. While the majority of its land area is used for residential purposes, a significant portion is used for industrial, commercial, institutional, and recreational purposes, strengthening its economic status as a highly urbanized center [29, 30]. Quezon City ranks first in the annual ranking of the Cities and Municipalities Competitive Index, which ranks all cities and municipalities in the Philippines based on economic dynamism, government efficiency, infrastructure, resilience, and innovation [31]. In terms of political administration, Quezon City is divided into 142 villages called “*barangays*” [30] (Fig. 2).

With a population of 2,960,048 residents based on the 2020 census, Quezon City is the most populated city in the Philippines [32]. However, its population growth rate from 2015 to 2020 was only 0.17% [32]. The population density is 17,300.11 persons per sq. km [32]. Five *barangays* in Quezon City were among the ten most populated *barangays* in the Philippines, which included Commonwealth (*N* = 213,229), Batasan Hills (*N* = 166,598), Payatas (*N* = 139,740), Holy Spirit (*N* = 111,901), and Pasong Tamo (*N* = 110,738) [32].

### Study design

This study employs an explanatory, multi-group comparison ecologic design, through which the dengue incidence will be compared across *barangays* of Quezon City and the potential factors influencing the magnitude will be explored [33].

### Data collection

Reported dengue cases in every quarter of each year from January 2019 to December 2022 were provided by the Quezon City Epidemiology and Surveillance Division (QCESD). The first quarter runs from January 1 to March 31 of each year, the second from April 1 to June 30, the third from July 1 to September 30, and the fourth from October 1 to December 31.In compliance with the Philippine Integrated Disease Surveillance and Response, all units of health service delivery designated as disease reporting units in Quezon City, i.e., *barangay* health stations, hospitals, and clinics, are mandated to routinely submit weekly reports of all dengue cases and COVID-19 cases to QCESD. The QCESD, in turn, submits its weekly summary to the regional epidemiology and surveillance unit. Aside from facilitating the collection of surveillance data, QCESD facilitates the collation, analysis, and interpretation of the data for the city [34].

The population data of each *barangay* for the national census years 2010 and 2015 were obtained from the Civil Registry Department of the Local Government of Quezon City. Population projections for 2019 to 2022 were estimated.

Buildings and facilities, and transportation network hubs were the pick-up land use that were obtained from satellite images of Sentinel-2 and OpenStreetMap [35, 36]. Normalized difference vegetation index (NDVI) was used as the indicator of greenness and surrounding greenness in this study. NDVI, by far, is the most commonly used index of green space in previous studies, which demonstrated its effectiveness in differentiating types of vegetation and estimating vegetation properties [24, 27, 37]. Calculated from the reflectance recorded with sensors in the visible red and NIR regions, its value may range from −1 to 1 [37]. Negative NDVI values are indicative of water bodies, while an NDVI of zero or nearly but above zero is suggestive of concrete surfaces such as rocks and sand, or concrete surfaces [37]. Positive NDVI, which are closer to the value of one, are indicative of vegetation such as shrubs, forests, and grasses [37].

### Data analysis

For each *barangay*, the quarterly incidence rate of dengue from 2019 to 2022 was expressed as the total number of cases times 10,000 and then divided by the total population in each year. The population densities of each *barangay* were calculated by dividing the population of each *barangay* by its respective land area, which was expressed in square kilometer units. All of these derived estimates were mapped using ArcGIS 10.8.2 (Environmental Systems Research Institute, Inc., Redlands, CA, USA). Local Moran’s I statistics was employed in the identification of dengue hotspots and cold spots performed at a 95% confidence level. It is a local indicator of spatial association (LISA) that could identify the exact location of spatial aggregations [38]. A positive I value is indicative of aggregation of *barangays* with high dengue incidence or aggregation forming a cluster of high incidence *barangays* (dengue hotspots)*,* or conglomeration of *barangays* with low dengue incidence forming a cluster of low incidence *barangays* (dengue cold spots) [39]. On the other hand, a negative I value indicates aggregation of *barangays* with different levels of dengue incidence, thus forming spatial outliers. A *p*-value of less than or equal to 0.05 is indicative of statistical significance of clustering [39].

Maps showing the spatial distribution of greenness and surrounding greenness were developed by mapping the NDVI and the surrounding NDVI of each *barangay* at 100 m, 500 m, and 1000 km [40]*.* Validation was done by overlapping and comparing the maps of buildings in OpenStreetMap with the satellite images in each barangay. The process showed that the presentation of buildings is similar to how those are presented in satellite imagery. For the purposes of this study, buildings with an area of 6 to 30 square meters were classified as small buildings, while those with an area of more than 30 square meters were classified as large buildings. The ratio of the sum of the areas of all small buildings expressed in square meters to the total sum of all buildings expressed in square meters was calculated in each *barangay* and was referred to as the small building ratio. A map of the spatial distribution of small building ratios was also developed. The number of transportation network hubs, which include terminals of all types of vehicles in Quezon City, was also mapped.

To determine the association of dengue cases with greenness, surrounding greenness, transportation network hub, small building ratio, and population density, a generalized linear model (GLM) was used with a negative binomial distribution and a log link function. STATA version 14 (StataCorp, College Station, TX, USA) was used in performing the analysis.

## Results

**Fig. 3:**
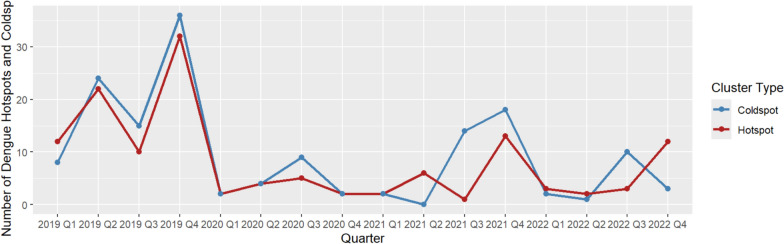
Quarterly trend of dengue hotspots and cold spots in Quezon City, the Philippines, 2019–2022

**Fig. 4:**
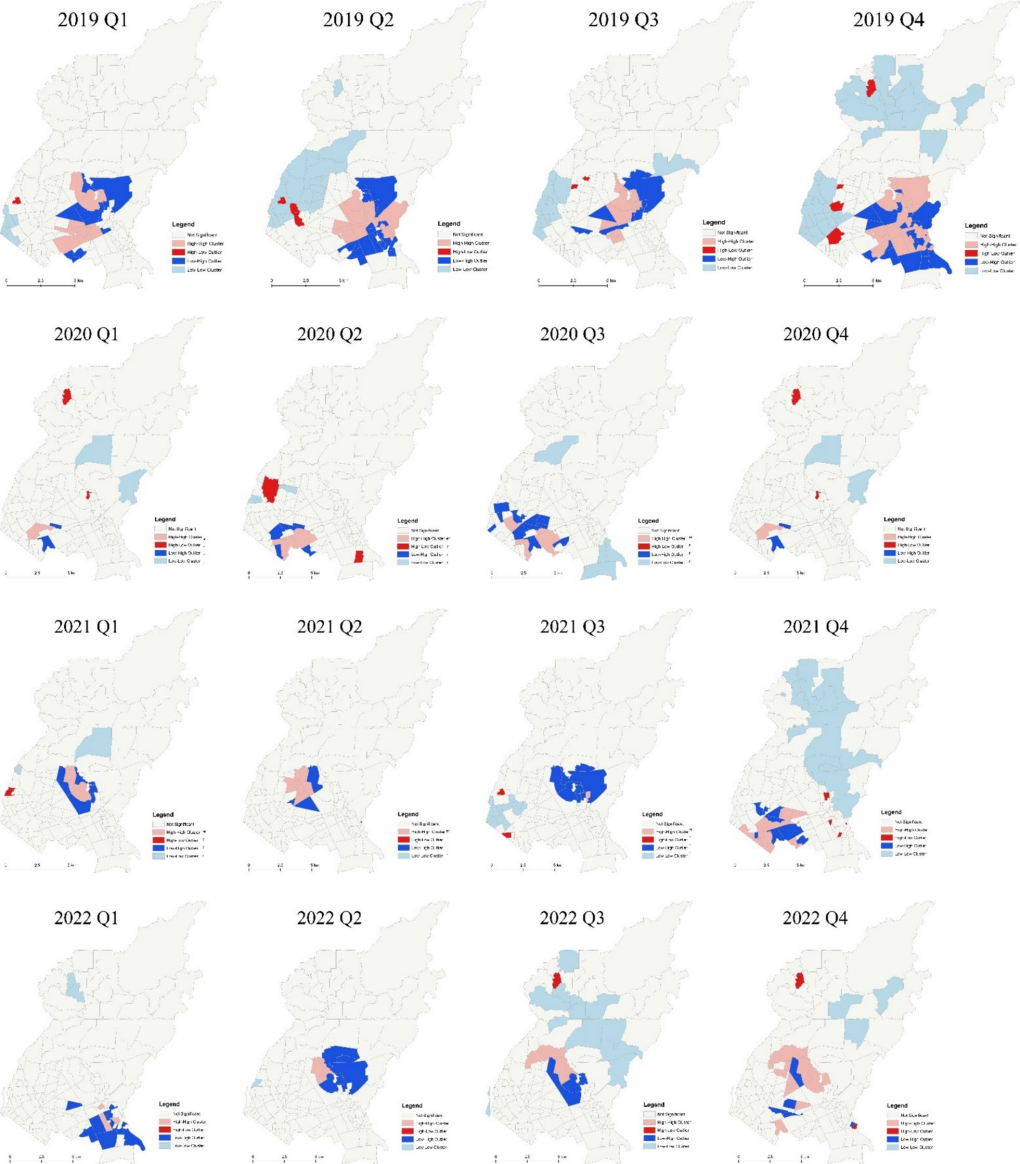
Quarterly maps of dengue hotspots in Quezon City from 2019 to 2022. **a** Greenness of *Barangays*, **b** surrounding greenness of *Barangays*

In 2019, the number of identified dengue hotspots was oscillating with the lowest count recorded in the third quarter and the highest count recorded in the last quarter (Fig. 3). Most of the dengue hotspots were *barangays* located in the southeastern portion of the city, and are mainly used for residential and institutional purposes, and some for commercial use (Fig. 4).

As the year transitioned to 2020, the number of dengue hotspots dropped by 16-fold to two *barangays* during the first quarter from 32 *barangays* during the last quarter of 2019. That was greatly below the number of identified dengue hotspots in the same quarter of the preceding year. The highest number of identified dengue hotspots in 2020 was five *barangays*, which was noted during the third quarter. This was half of the number of dengue hotspots that were identified in the preceding year (Fig. 3). Most of the dengue hotspots that were identified this year were located in the southwestern part of Quezon City (Fig. 4). These *barangays* were mostly residential areas.

During the first quarter of 2021, the number of identified dengue hotspots remained the same as the number of dengue hotspots during the last quarter preceding year. However, these two *barangays*, which were located near the center portion of the city terrain and are mainly residential in nature, were different from the previous two dengue hotspots (Fig. 4). The number of dengue hotspots had increased by almost sevenfold during the last quarter of the year (Fig. 3). These *barangays* are mainly residential areas.

From 13 dengue hotspots in the last quarter of 2021, the number of dengue hotspots during the first quarter of 2022 dropped to only three *barangays*. This gradually increased until a spike in the number of dengue hotspots in the last quarter of the year, which was equal to pre-pandemic magnitude (Fig. 3). The identified hotspots during this year, mostly located in the southern part of Quezon City, were mainly residential and institutional areas with some commercial areas (Fig. 4).

**Fig. 5:**
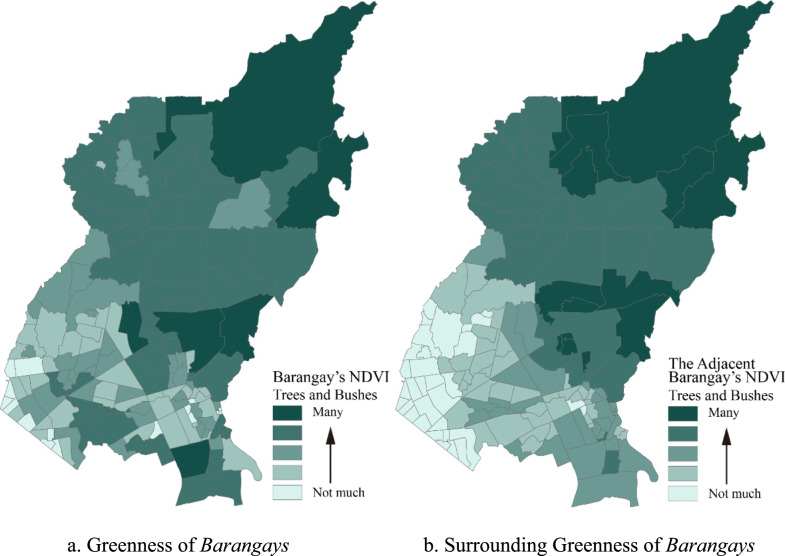
Spatial distribution of greenness and surrounding greenness of *Barangays* in Quezon City. **a** Greenness of *Barangays,* **b** surrounding greenness of *Barangays*

*Barangays* in the northern part of the city, which were relatively larger in terms of land areas, had more trees and bushes, as indicated by their greenness and surrounding greenness. The smaller *barangays* in the southwestern part of the city, which were adjacent to the City of Manila, had lesser vegetation indices (Fig. 5). These areas were composed mainly of residential lands and some industrial areas.

**Fig. 6:**
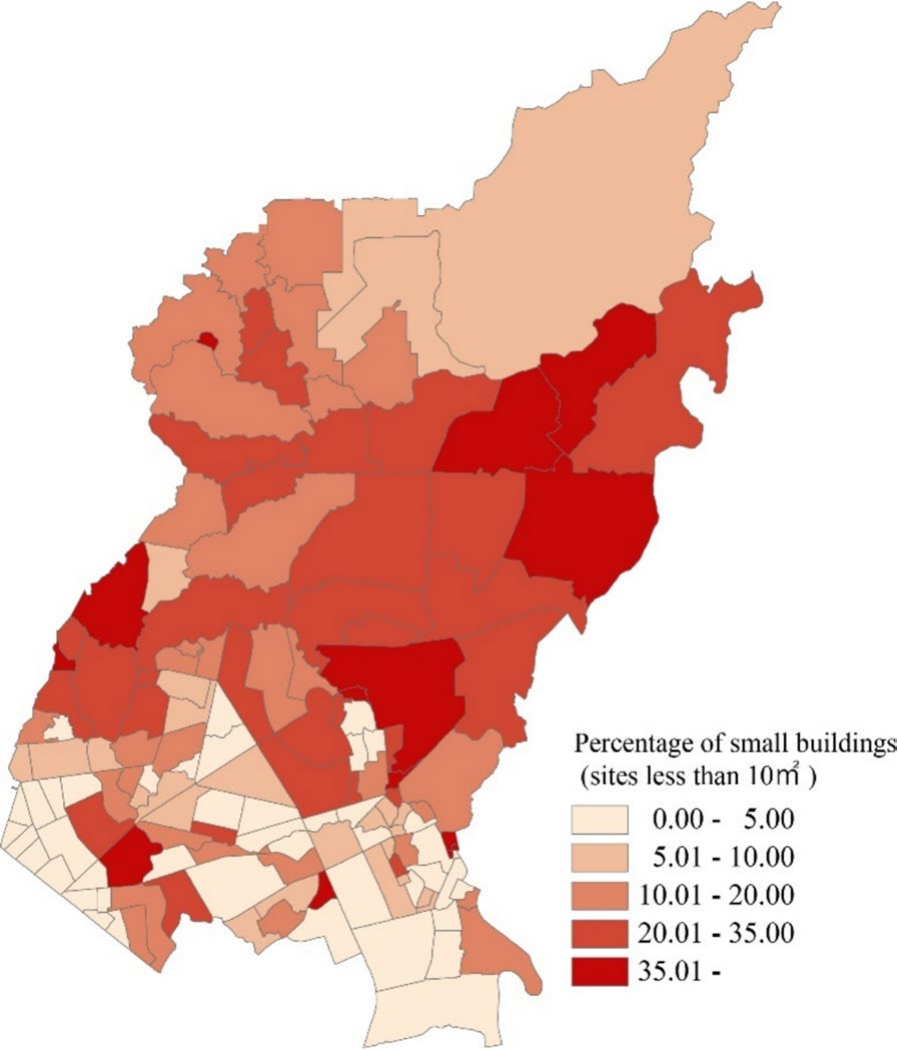
Spatial distribution of small buildings in *Barangays* in Quezon City

**Fig. 7:**
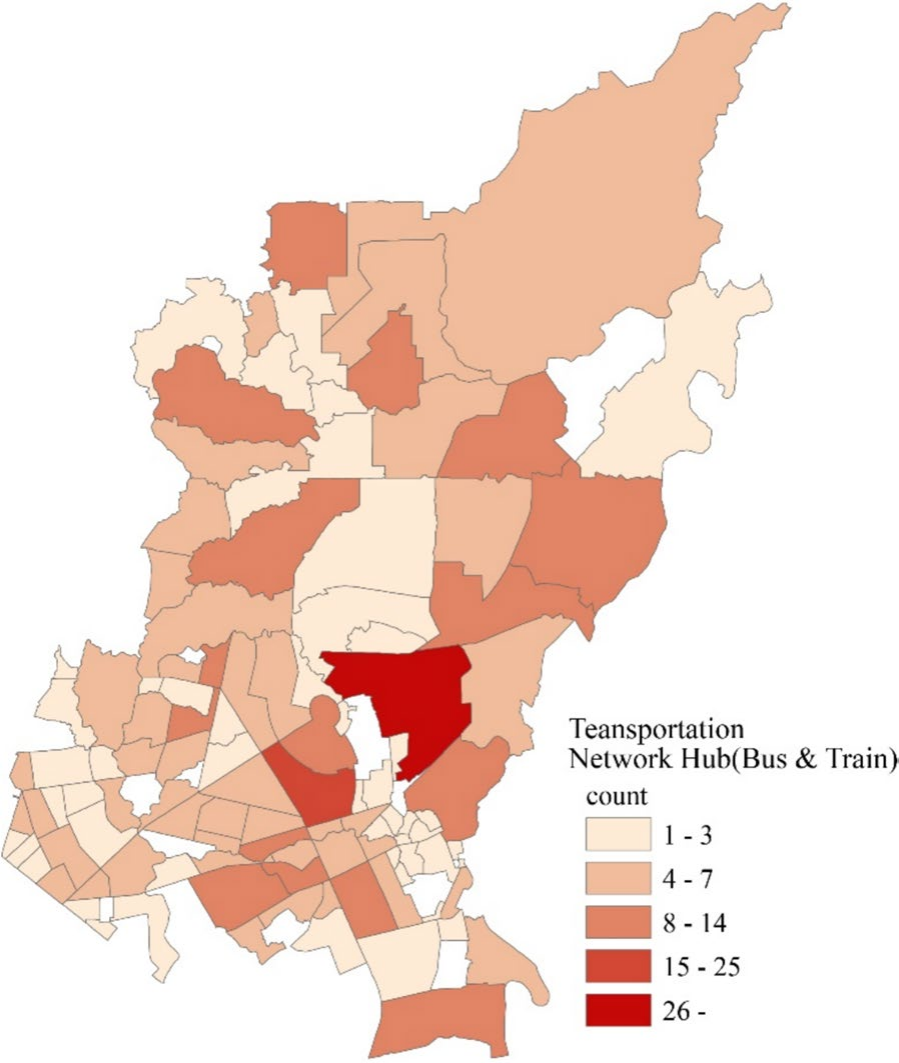
Spatial distribution of transportation network hubs (stops) in *Barangays* in Quezon City

On the other hand, small buildings were not that prominent in small *barangays*. There are more small buildings in relatively larger *barangays* (Fig. 6). The transportation network hubs were also heterogeneously distributed among the *barangays* (Fig. 7).

Surrounding greenness at 1 km of each *barangay* was found to be negatively associated with dengue cases. A unit increase in the surrounding NDVI can lead to a decrease in dengue cases by 8.49 units. On the other hand, small building ratio and the density of transportation network hubs were found to be positively associated with dengue cases. A unit increase in small building ratio can result in a 0.04 unit increase in dengue cases. On the other hand, a unit increase in the density of transportation network hubs can yield a 0.072 unit increase in dengue cases (Table 1).

**Table 1:** Result of generalized linear model in determining associated environmental and demographic factors with dengue in Quezon City

Factors	Dengue cases		
	Coefficient	95% CI	*p*-value
NDVI	1.14	−1.30 to 3.58	0.359
Surrounding NDVI 1 km	−8.49	−16.51 to −0.46	0.038*
Surrounding NDVI 500 m	5.78	−3.74 to 15.29	0.234
Surrounding NDVI 100 m	−2.11	−6.14 to 1.93	0.306
Small building ratio	0.04	0.02 to 0.06	<0.0001*
Transportation network hubs	0.072	0.02 to 0.12	0.004*
Population density	−0.0000059	−0.0000153 to 0.000006	0.218

*Statistically significant

## Discussion

In the current study, the variations in the quarterly distribution of reported dengue cases in Quezon City from 2019 to 2022 were investigated through hot spot identification. The results revealed that the incidence rates of dengue across *barangays* were spatially heterogeneous and the dengue hotspots were unstable as they vary across each quarter of each year. With the advent of the COVID-19 pandemic, the number of dengue hotspots tremendously decreased. A resurgence was noted in the last quarter of 2022. Reported dengue cases also tend to reach a peak in the last quarter of every year, except in 2020. Proximity to surrounding greenness (NDVI 1 km), small building ratio, and presence of transportation network hubs were also found to be significantly associated with dengue hotspots.

The apparent decline in the number of dengue hotspots in Quezon City from 2019 to 2022 is a spinoff of the reduced number of reported dengue cases. Such a trend was also observed in a study in Sri Lanka that reported an overall 88% reduction in dengue risk during the community lockdown [41]. The Philippine government had also resorted to this strict measure of mitigating COVID-19 transmission. On 16 March 16, 2020, NCR and the rest of Luzon was placed under a total lockdown, limiting people’s mobility within and outside the region and hampering regular access to essential activities [42].

Some variations in the community lockdowns were implemented in August 2020 and March 2021 [43, 44]. To promote economic activities amid the reduction in COVID-19 cases during the Christmas season, the government allowed 50 and 70% occupancy for indoor and outdoor venues, respectively, in NCR in December 2021 [45]. This promoted mobility and could explain the rise in dengue cases and the hotspots in the latter part of the year. A similar finding was observed in a 2019 study in China [46]. The increase in mobility facilitated the spread of the virus among travelers and facilitated the establishment of nearby dengue hotspots during the last quarter of 2021. It was also noted that the rise in the number of cases during the last months of the year can be explained by the high relative humidity experienced in this period [47]. The number of COVID-19 cases also rose, leading to the reimposition of community restrictions in January 2022, which equated to a reduction again in dengue hotspots in the succeeding quarters [48].

The public health and social measures such as community quarantine, school closures, and lockdowns, imposed by the government to curb the surge of COVID-19 cases restricted human mobility, which could have negatively affected dengue transmission. But with the shift of the government’s focus on strengthening the COVID-19 response, the disruption in the delivery of essential health services could have also affected disease surveillance, which may have resulted in the underreporting of diseases including dengue. The decline in reported dengue cases may also be attributed to reporting hesitancy due to people’s fear of acquiring COVID-19 upon visiting a health facility. The anticipated stigma associated with COVID-19 and the fear of being secluded in isolation and quarantine facilities might have swayed people not to disclose their signs and symptoms and avoid testing, leading to poor case detection of both COVID-19 and dengue.

Time series plot showed that dengue hotspots tend to peak during the last quarters of every year, except during the height of the pandemic in 2020. This is an expected finding as a previous study of dengue in Quezon City had shown that seasonality is inherent in the time series structure of dengue cases and tends to peak during the wet season [49]. It was found that meteorological factors such as rainfall, humidity, and temperature correlated with dengue cases [50].

Surrounding greenness (surrounding NDVI at 1 km) was found to be negatively associated with dengue cases through GLM. This is similar to the results of an ecological study done in 2021 in Belo Horizonte, Brazil [24]. In another study in Taipei published in 2021, disaggregated data among the types of greenspaces revealed a negative correlation for farms, forests, and grasslands [27]. The association between surrounding greenness and dengue can be explained by the thriving of dengue vectors in vegetation. In QC, a previous study in 2022 on mosquito populations proved the presence of *Ae. aegypti* and *Ae. albopictus* in selected areas through ovitraps [51]. Among the dengue vectors Aedes species, *Aedes aegypti* has been said to be the main vector in Manila, Philippines, with the co-existence of a small proportion of *Aedes albopictus* [52], and this vector co-existence situation may accelerate dengue outbreak [53]. These two vectors are similar in morphology (body size and color pattern), flying range (unable to fly long distance), biting time (day time), and breeding site (any small artificial or natural containers) [54, 55]. However, their habitat is slightly different as *Ae. aegypti* are likely to live close to human houses, whereas *Ae. albopictus* tend to be found in parks, planted yards, and bush [56]. *Ae. aegypti* prefer to live indoors and lay eggs mostly in artificial containers found both inside and outside the house, while *Ae. albopictus* were frequently found outdoors and lay eggs in artificial or natural containers also outdoors, such as coconut husks, coconut floral spathes, flower pots in cemeteries, etc. [52, 57]. *Ae. aegypti* almost always feed on humans, while *Ae. albopictus* feed on humans but also other mammalian hosts, such as cats [58, 59]. Both *Ae. aegypti* and *Ae. albopictus* are susceptible to DENV; however, the infectivity of *Ae. aegypti* is reportedly higher than *Ae. albopictus* [60, 61], and it seems like only *Ae. aegypti* has an important role in having a cluster of dengue cases. However, a previous study in Taiwan discussed that an outbreak may be initiated by *Ae. aegypti* and be expanded by *Ae. albopictus* [53]. The southern part of Taiwan, where both species occur, has experienced major epidemics. It is therefore likely that the co-existence of the two vectors, rather than the presence of *Ae. aegypti* alone, has influenced outbreaks in recent years, which may be consistent with the results of this study.

Results of the GLM showed that the density of transportation network hubs or terminals in Quezon City was positively associated with dengue cases. This is consistent with the findings in a previous study in Bangkok, which found that the density of public transportation stops is higher in the identified dengue hotspots, and a small but positive association with dengue cases was seen in their multivariable risk factor model [17].

**Fig. 8:**
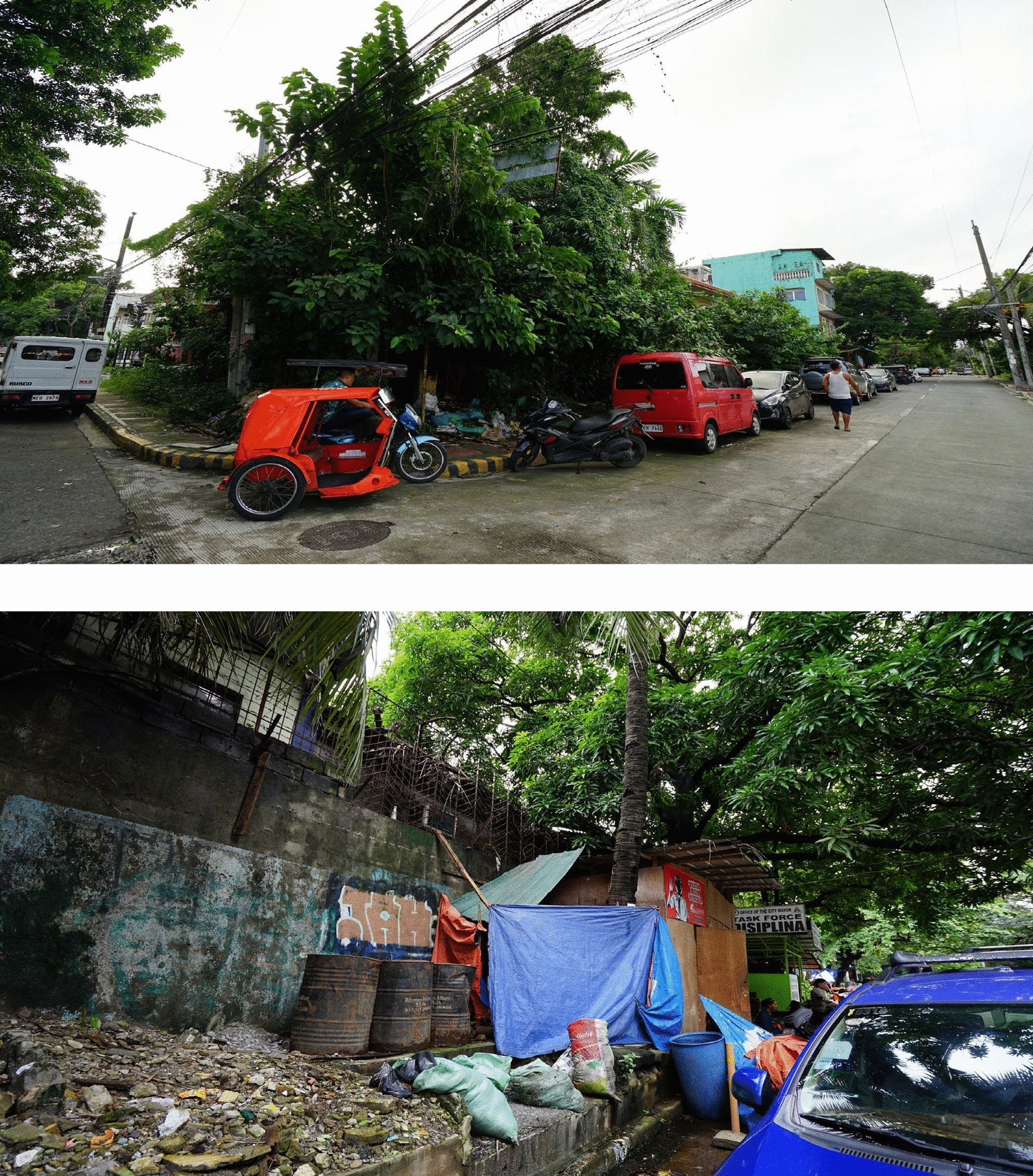
Neighboring houses and shacks in Quezon City surrounded by trees and shrubs. August 4, 2023

Another finding of the current study was the positive association between dengue cases and the small building ratio, which pertains to the ratio of the sum of the areas of all small buildings expressed in square meters to the total sum of all buildings expressed in square meters in each *barangay*. Previous studies had explored the impact of patterns of urban housing with dengue cases [24, 62]. In a study in Brazil, the association of building height with dengue incidence was explored and was found to be negative but not significantly associated [24]. On the other hand, a study in Singapore showed that building area is associated with dengue incidence. Since outdoor breeding drains of *A. aegypti* were found to be clustering around the sub-area of low-rise houses, the incidence of dengue was found to be higher in this subarea than in the subarea of high-rise buildings [62]. The findings of this study are consistent with the findings of the current study, except that the interpretation of building area in the previous study took also into account the height of the building, while the interpretation of area in the current study corresponds to the planar surface area occupied by the building. Figure 8 shows samples of houses surrounded by bushes in *barangays* detected as dengue hotspots. Small shacks, which also serve as residence for informal settlers, were also seen along the vicinity of small, densely built-up areas of houses that are surrounded by vegetation. The interiors of these houses and shacks are assumed to be a habitat for *Ae. aegypti*.

**Fig. 9:**
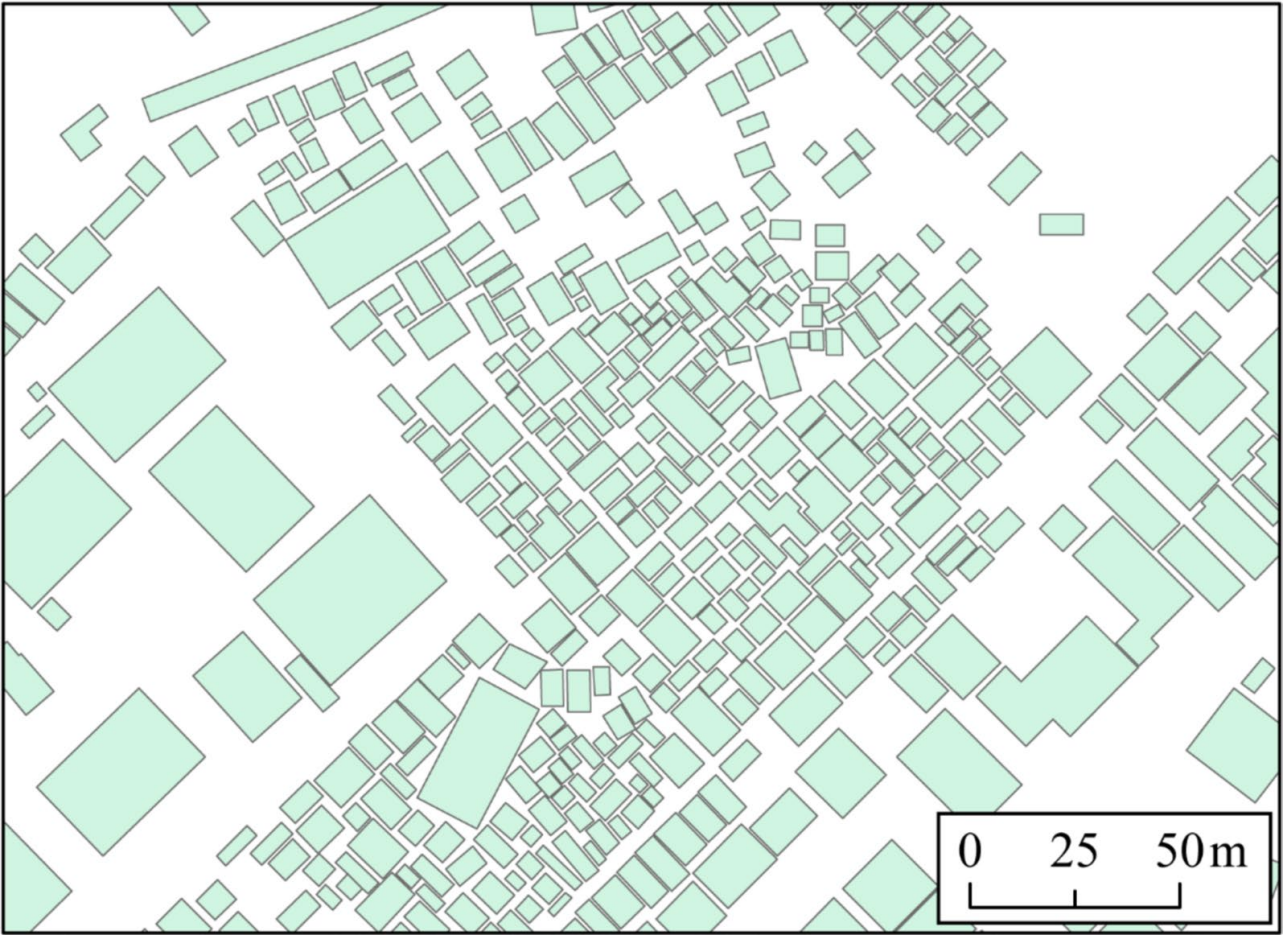
Sample map of buildings in Quezon City. OpenStreetMap. 2023

**Fig. 10:**
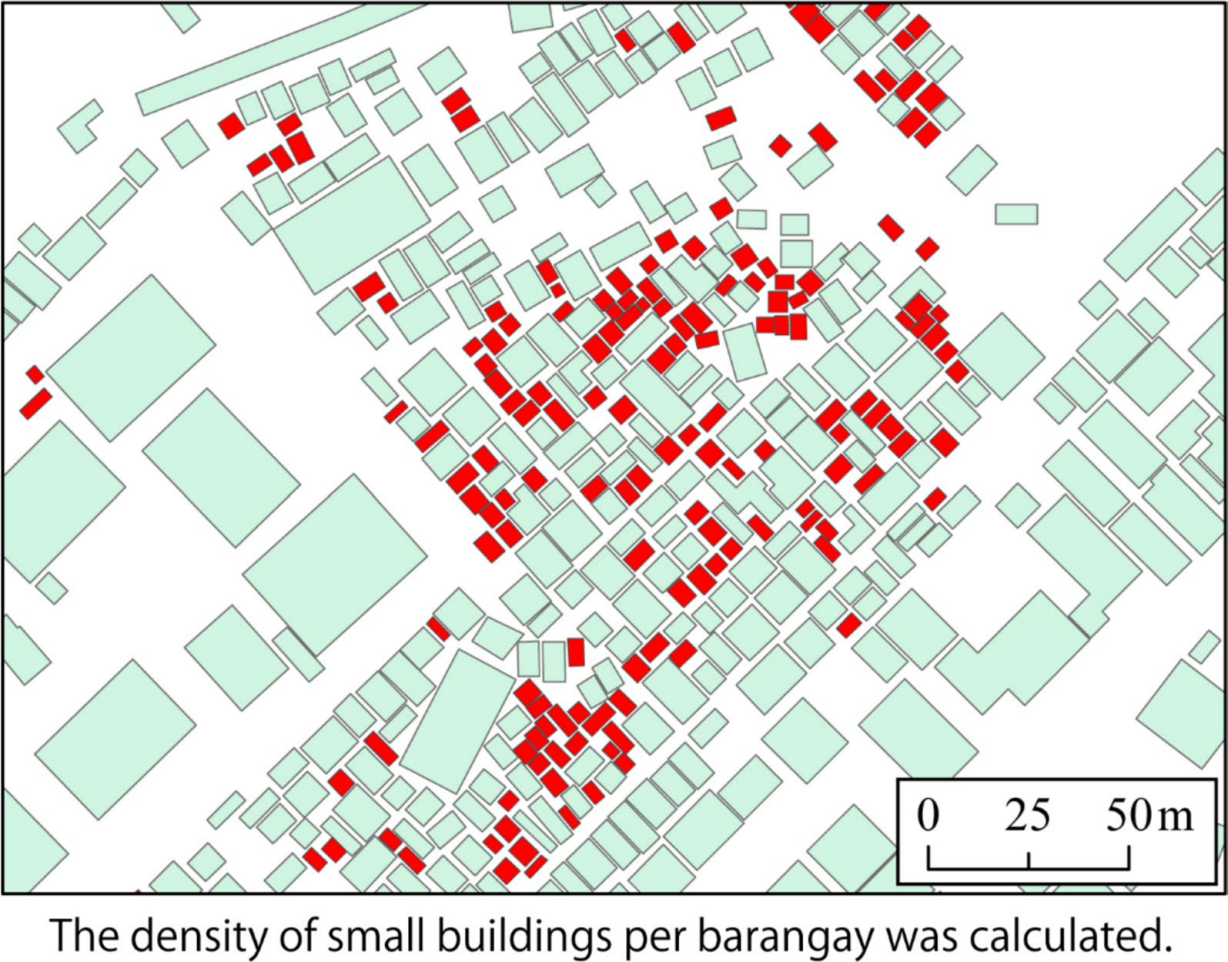
Spatial distribution of small buildings in Quezon City. OpenStreetMap. 2023. The *red colored polygons* are classified as small buildings with an area ranging from 6 to 30 square meters. The *green polygons* are those buildings with an area greater than 30 sq. m

It can be appreciated from the maps that the identified dengue hotspots were areas with high concentrations of small buildings and surrounding green areas. Figure 9 shows that there were gaps between large commercial and public buildings. These areas are not only homes for well-maintained parks, but also for many unmaintained and neglected bushes, where mosquito vectors could be thriving. Figure 10 presents the result of overlapping the green areas revealed by satellite imagery with the map showing small houses. This clearly shows the formation of bushes around small houses.

Free environmental data from OpenStreetMap (OSM) and images from satellite imagery were utilized in this study. Their use in the prevention and control of dengue has already been demonstrated in Indonesia and Brazil. In Brazil, OSM data were used in a project to identify mosquito breeding sites in the urban area of Rio de Janeiro and to design the appropriate response. Once the location of puddles and rubbish dumps were identified, clean-up and awareness-raising activities were carried out to prevent mosquito breeding [63]. In a research project in Indonesia, the OSM was linked to a mobile phone application. Residents reported outbreaks of dengue fever and the source of mosquitoes to the application, and the data were fed into the OSM, allowing real-time monitoring and response [64]. This study was able to derive relevant findings by overlaying high-resolution sanitary images on OSM. OSM is free and has high accuracy, especially in urban areas [65]. Accuracy is also increasing in low- and middle-income countries, particularly in Asia, and is high in cities such as Manila, Jakarta, Singapore, and Bangkok, where dengue fever is hyperendemic. On the other hand, the availability of high-resolution sanitary images is weather-dependent. Free ones are currently limited, as they are also useful for commercial and military applications. If the use of these high-resolution maps is promoted to strengthen public health activities in the future, they could also be applied to dengue fever control by complementing the use of OSM. Yet a process to validate this crowdsourced data should be in place to balance accuracy with convenience and utility.

The results of this study should be interpreted in light of the following limitations. The reported dengue cases are only either suspected or probable, and not confirmed cases. There could have been an underestimation of dengue incidence per *barangay*, but the effect on the number of hotspots could not be predicted. In the GLM, the input data included only the latest NDVI in 2021 following the assumption that there was not much change in vegetation in the past four years. The input data for dengue cases covered the period from 2019 to 2022. Poor surveillance of dengue cases during the height of the COVID-19 pandemic could have affected the general trend. In spite of these limitations, the findings of this study will be useful for designing tailored-fit interventions for the situation of Quezon City. As part of delimiting the scope of the study, meteorological factors such as surface temperature, rainfall, vector indicators such as mosquito density, and water access were not included in the GLM. The association of these factors can be further explored in future studies. Likewise, the current regression method that was applied did not take into account spatial weights. Hence, there is an avenue for this approach to be applied in future studies.

## Conclusion

This study found that incidence rates of dengue across *barangays* in Quezon City were spatially heterogeneous, and the dengue hotspots were unstable as they varied across each quarter of each year. With the advent of the COVID-19 pandemic in 2020, the number of dengue hotspots tremendously decreased. A resurgence was noted in the last quarters of 2021 and 2022. Proximity to surrounding greenness (NDVI 1 km), small building ratio, and presence of transportation network hubs were found to be significantly associated with dengue hotspots. Dengue prevention strategies such as search-and-destroy activities for breeding sites are suggested to be implemented regularly in these areas. The study also recommends taking into consideration the anticipated increase in the dengue hotspots, i.e., before the last quarter of the year, and the significant environmental factors in the development and timing of health promotion and education campaigns. Specifically, health education activities to prevent and control dengue should be continued all year round but can be further intensified before the anticipated peak in the number of cases, particularly for high-risk areas. The study also recommends performing spatial analysis using monthly dengue cases. Spatial analysis of the association of dengue hotspots with other demographic and social determinants of health such as education, income, and occupation can be explored in future studies. Future researchers may also look into the specific types of greenspaces present and their corresponding association with dengue incidence.

## Supplementary Information

Additional file 1.

## Data Availability

No datasets were generated or analyzed during the current study.

## Abbreviations

COVID-19: Coronavirus disease 2019
NCR: National Capital Region
QCESD: The Quezon City Epidemiology and Surveillance Division
GLM: Generalized Linear Model
