# Supplementary material for: Pre- and post-COVID-19 pandemic identification of dengue hotspots and exploration of population and environmental determinants of dengue in Quezon City, Philippines
Source: Trop Med Health. 2025 Aug 14;53:109. doi: 10.1186/s41182-025-00789-3 (PMC12351799; doi:10.1186/s41182-025-00789-3)
Supplement: Supplementary file 1 — Additional file 1. [file 41182_2025_789_MOESM1_ESM.pdf]

No	Barangay Name
1	Alicia
2	Amihan
3	Apolonio Samson
4	Aurora
5	Baesa
6	Bagbag
7	Bagong Lipunan Ng Crame
8	Bagong Pag-Asa
9	Bagong Silangan
10	Bagumbayan
11	Bagumbuhay
12	Bahay Toro
13	Balingasa
14	Balong Bato
15	Batasan Hills
16	Bayanihan
17	Blue Ridge A
18	Blue Ridge B
19	Botocan
20	Bungad
21	Camp Aguinaldo
22	Capri
23	Central
24	Claro
25	Commonwealth
26	Constitution Hills
27	Culiat
28	Damar
29	Damayan
30	Damayang Lagi
31	Del Monte
32	Dioquino Zobel
34	Doña Imelda
35	Doña Josefa
33	Don Manuel
36	Duyan-Duyan
37	E. Rodriguez
38	East Kamias
39	Escopa I
40	Escopa II
41	Escopa III
42	Escopa IV
43	Fairview
44	Gulod
45	Holy Spirit
46	Horseshoe
47	Immaculate Concepcion
48	Kaligayahan

No	Barangay Name
49	Kalusugan
50	Kamuning
51	Katipunan
52	Kaunlaran
53	Kristong Hari
54	Krus Na Ligas
55	Laging Handa
56	Libis
57	Lourdes
58	Loyola Heights
59	Maharlika
60	Malaya
61	Mangga
62	Manresa
63	Mariana
64	Mariblo
65	Marilag
66	Masagana
67	Masambong
68	Matalahib
69	Matandang Balara
70	Milagrosa
74	New Era
71	N.S. Amoranto
72	Nagkaisang Nayon
73	Nayong Kanluran
75	Novaliches Proper
76	Obrero
77	Old Capitol Site
78	Paang Bundok
79	Pag-Ibig Sa Nayon
80	Paligsahan
81	Paltok
82	Pansol
83	Paraiso
84	Pasong Putik Proper
85	Pasong Tamo
86	Payatas
87	Phil-Am
88	Pinagkaisahan
89	Pinyahan
90	Project 6
91	Quirino 2-A
92	Quirino 2-B
93	Quirino 2-C
94	Quirino 3-A
95	Ramon Magsaysay
96	Roxas

No	Barangay Name
97	Sacred Heart
98	Saint Ignatius
99	Saint Peter
100	Salvacion
101	San Agustin
102	San Antonio
103	San Bartolome
105	San Isidro Labrador
104	San Isidro
106	San Jose
107	San Martin de Porres
108	Quezon Memorial Circle
109	San Roque
110	San Vicente
111	Sangandaan
112	Santa Cruz
113	Santa Lucia
114	Santa Monica
115	Santa Teresita
116	Santo Cristo
117	Santo Niño
118	Santol
119	Sauyo
120	Sienna
121	Sikatuna Village
122	Silangan
123	Socorro
124	South Triangle
125	Tagumpay
126	Talayan
127	Talipapa
128	Tandang Sora
129	Tatalon
130	Teachers Village East
131	Teachers Village West
132	U.P. Campus
133	U.P. Village
134	Ugong Norte
135	Unang Sigaw
136	Valencia
137	Vasra
138	Veterans Village
139	Villa Maria Clara
140	West Kamias
141	West Triangle
142	White Plains
